# An Analysis of 30-Day in-Hospital Trauma Mortality in Four Urban University Hospitals Using the Australia India Trauma Registry

**DOI:** 10.1007/s00268-020-05805-7

**Published:** 2020-10-21

**Authors:** Prashant Bhandarkar, Priti Patil, Kapil Dev Soni, Gerard M. O’Reilly, Satish Dharap, Joseph Mathew, Naveen Sharma, Bhakti Sarang, Anita Gadgil, Nobhojit Roy, Australia-India Trauma System Collaboration

**Affiliations:** 1 Trauma Research Group, WHO Collaborating Centre for Research in Surgical Care Delivery in LMICs, Mumbai, India; 2 Critical and Intensive Care, JPN Apex Trauma Centre, AIIMS, New Delhi, India; 3 National Trauma Research Institute, The Alfred, Melbourne, Australia; 4 School of Health System Studies, Tata Institute of Social Sciences, Mumbai, India; 5 Topiwala National Medical College & B.Y.L. Nair Ch. Hospital, Mumbai, India; 6 Department of Surgery, All India Institute of Medical Sciences (AIIMS), Jodhpur, India; 7 Department of Global Public Health, Karolinska Institutet, 171 77 Stockholm, Sweden; 8 School of Public Health and Preventive Medicine, Monash University, Melbourne, Vic Australia

## Abstract

**Background:**

India has one-sixth (16%) of the world’s population but more than one-fifth (21%) of the world’s injury mortality. A trauma registry established by the Australia India Trauma Systems Collaboration (AITSC) Project was utilized to study 30-day in-hospital trauma mortality at high-volume Indian hospitals.

**Methods:**

The AITSC Project collected data prospectively between April 2016 and March 2018 at four Indian university hospitals in New Delhi, Mumbai, and Ahmedabad. Patients admitted with an injury mechanism of road or rail-related injury, fall, assault, or burns were included. The associations between demographic, physiological on-admission vitals, and process-of-care parameters with early (0–24 h), delayed (1–7 days), and late (8–30 days) in-hospital trauma mortality were analyzed.

**Results:**

Of 9354 patients in the AITSC registry, 8606 were subjected to analysis. The 30-day mortality was 12.4% among all trauma victims. Early (24-h) mortality was 1.9%, delayed (1–7 days) mortality was 7.3%, and late (8–30 days) mortality was 3.2%. Abnormal physiological parameters such as a low SBP, SpO2, and GCS and high HR and RR were observed among non-survivors. Early initiation of trauma assessment and monitoring on arrival was an important process of care indicator for predicting 30-day survival.

**Conclusions:**

One in ten admitted trauma patients (12.4%) died in urban trauma centers in India. More than half of the trauma deaths were delayed, beyond 24 h but within one week following injury. On-admission physiological vital signs remain a valid predictor of early 24-h trauma mortality.

## Introduction

Globally, injuries claim more lives than HIV/AIDS, TB, and malaria together [1]. India has one-sixth (16%) of the world’s population but over one-fifth (21%) of the world’s injury mortality. There are more than a million people who die following injury each year in India [1, 2]. Globally, age-standardized death rates for transport injury have decreased since the 1990s. However, India’s injury-related death rates have been on the rise [3].

In 2011, the WHO declared a Decade of Action for Road Safety, [4] to implement pre-hospital and in-hospital trauma survival strategies. The Global Road Safety Report recommended the 30-day fatality criteria (dying within 30 days of injury) as a standard to compare post-crash outcomes across trauma centers, within and among nations [5]. In previous studies, in-hospital trauma mortality in Indian hospitals was double that of high-income countries (HIC) [6]. Half of the trauma deaths in India occur at the scene of the injury or on the way to hospital (second delay), while the remaining half of trauma deaths occur following arrival at the hospital (second or third delay) [7]. It has been estimated that by providing the appropriate and timely trauma care in hospitals which exists in many HICs in low-to-middle-income countries (LMICs) settings, two million deaths might be averted annually [8].

Data from the trauma registry contain on-admission vital signs necessary to inform the patient’s physiological scoring systems. Such details have been used to predict early in-hospital mortality [3]. Anatomical scoring systems and injury scoring systems aid in standardizing comparisons across trauma centers and may inform improvements in the quality of trauma care practices across trauma centers. However, despite the large burden of injuries in India, the literature on severity-adjusted 30-day mortality remains sparse. Previously in 2016, a large multi-institutional study systematically documented 30-day in-hospital trauma mortality in 11,202 patients and found that one in five admitted patients died in India’s major urban trauma centers [6]. In this study, we used another extensive trauma registry produced through a bilateral initiative between the Indian Government and the Australian Government called the Australia India Trauma Systems Collaboration (AITSC) [9]. This study aimed to describe the 30-day in-hospital trauma mortality in high-volume trauma units within four university hospitals in urban India. Secondarily, we assessed the associations between demographic, physiological, and process-of-care factors with early (0–24 h), delayed (1–7 days), and late (8–30 days) in-hospital trauma mortality while adjusting for injury severity.

## Materials and methods

### Study background

The AITSC Project created a trauma registry, using a prospective multicenter observational cohort at four urban public tertiary care hospitals across three Indian cities. This study is a retrospective analysis of the AITSC registry data. The data were extracted from the registry for patients that presented from April 2016 to March 2018. The AITSC partnership was led by the National Trauma Research Institute (NTRI), a department of Monash University and Alfred Health, and the Jai Prakash Narayan Apex Trauma Centre, All India Institute of Medical Sciences (AIIMS), Delhi.

### Study setting and participating hospitals

The AITSC registry involved four major Indian trauma hospitals—the Jai Prakash Narayan Apex Trauma Centre (JPNATC), New Delhi; the Lokmanya Tilak Municipal General Hospital(LTMGH), Mumbai; the Sheth Vadilal Sarabhai (VS) General Hospital, Ahmedabad; and the Guru Teg Bahadur (GTB) Hospital, Delhi. Each of the participating hospitals is a referral hospital for tertiary trauma care for neighboring suburbs and states.

JPNATC is a standalone trauma center at All India Institutes of Medical Sciences (AIIMS), New Delhi. There are 2362 beds at AIIMS altogether, of which 180 trauma dedicated beds function at JPNATC with advanced trauma care facilities. LTMGH is a 1416-bedded general hospital in Mumbai with 25 trauma intensive care beds. GTBH is a 2500-bedded hospital in New Delhi, with 7 main ICU and 7 neuro ICU beds. In comparison, VSH is a 1115-bedded hospital in Ahmedabad city with 15 ICU beds. All the study centers are public university hospitals across metropolitan cities and cater to the various services, including trauma care.

### Eligibility criteria

**Fig. 1:**
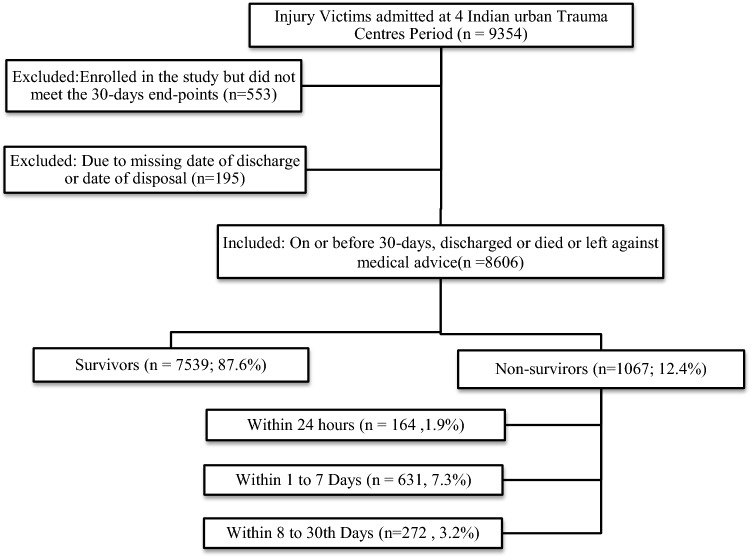
Recruitment of trauma patients for the AITSC trauma registry

All patients presenting to the emergency department with a history of injury and with a mechanism of road traffic, railway, fall or assault and admitted to the hospital were included. Patients who were dead on arrival were not included. Patients included in the data analysis were those who met the primary end-points of (1) death, (2) discharge, or (3) 30-day in-hospital stay. Those who could not be observed for a full 30-day period before the study ended were excluded from the analysis. Patients with the missing records of admission or hospital disposition dates were excluded. The recruitment algorithm is displayed in Fig. 1.

### Variables

Our primary outcome measure was mortality within 30 days of admission. Time to the primary outcome was further categorized as early (0–24 h), delayed (1–7 days), and late (> 7 days) [10]. Study parameters included age, sex, mode of transport, injury mechanism, and Injury Severity Score (ISS). Physiological parameters observed were on-admission systolic blood pressure (SBP), Glasgow Coma Scale (GCS), respiratory rate (RR), oxygen saturation level (SpO2), and heart rate (HR). This was consistent with the recommended process-of-care measurement for administering the local health service delivery as per the WHO recommendation [11]. Survival was measured in relation to the time of injury to first vital sign recording, as this time period represented the second delay in ‘reaching care’ (injury to hospital) combined with the third delay in ‘receiving care’ (hospital arrival to admission) [6, 12].

### Statistical methods

Data were analyzed using R Studio for Windows (version 3.1.7, R Working Group) and SPSS Version 24 (SPSS Inc., IBM Corporation, Chicago). An independent sample t-test was used to examine the differences between continuous variables. Kaplan–Meier survival analyses were performed across age groups, SBP, GCS, and mechanism of injury. Normally distributed numerical variables were reported as mean and standard deviation. Ordinal and skewed numerical variables were displayed using median with interquartile range. Nominal variables were represented in absolute numbers and group percentages. A *p* value below 0.05 was considered statistically significant.

Ethics approval for these studies was granted by each hospital’s human research ethics committee site AIIMS (IEC/NP-327/2013); LTMG-IEC/83/14; VS-approved 13/11/2013; GTB-approved 12/2/2015); and the individual trauma patient on-admission consent process was waived for observational data. In Australia, the AITSC program of work was approved by the Alfred Hospital Ethics Committee (Project 245/17) and the Monash University Human Research Ethics Committee (CF16/1814 – 2,016,000,929).

## Results

There were 9354 patients in the AITSC registry, and 8606 were eligible for analysis after applying the inclusion–exclusion criteria for this study (Fig. 1). The 30-day mortality was 12.4% among all trauma victims. Early (24-h) mortality was 1.9%, delayed (1–7 days) mortality was 7.3%, and late (8–30 days) mortality was 3.2%. There was no statistically significant difference in mortality between males and females (*p* = 0.30). However, male trauma patients were 81.7% of the total trauma victims.

**Fig. 2:**
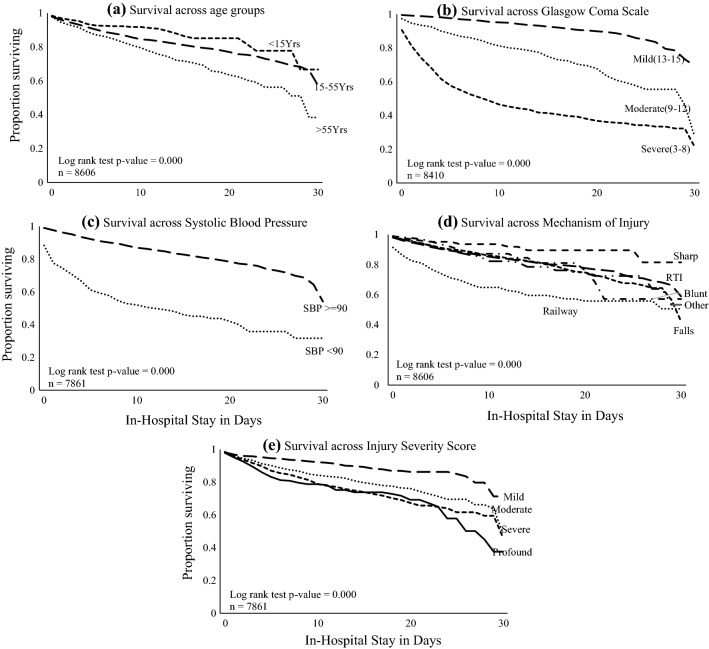
Kaplan–Meier survival graph (a) age groups, (b) on-admission Glasgow Coma Scale scores, (c) as per systolic blood pressure, (d) mechanism of injury, and (e) Injury Severity Score

Table 1 and Fig. 2 display the univariable unadjusted analysis results, comparing those who died in the hospital within 30 days versus those who survived. The mean age (SD) of those survivors and non-survivors was 30.3 (18.2) and 36.9 (18.9) years, respectively. The age distribution of survivors and non-survivors was approximately normal. For the age group of > 55 years, mortality was 19.6% compared to 6.6% for the youngest age group < 15 Years (*p* value < 0.01). Regarding GCS, a lower score was associated with a significantly higher mortality (*p* value < 0.001).

**Table 1:** Univariable analysis of trauma patients admitted to urban Indian hospitals

Variables	Overall	Survival	Death
	*n* = 8606	*n* = 7539	*n* = 1067
Age (Mean (SD))	31.1 (18.4)	30.3 (18.2)	36.9 (18.9)
Female	1574 (18.3%)	1391 (18.5%)	183 (17.2%)
Male	7031 (81.7%)	6147 (81.5%)	884 (82.8%)
Mechanism of injury (column % with 95% CI)			
RTI	46.9	46.6% (45.5–47.7)	48.7% (45.7–51.7)
Falls	32.1	32.5% (31.4–33.5)	28.8% (26.1–31.5)
Assault penetrating	5.6	6.1% (5.6–6.6)	2.3% (1.4–3.1)
Assault blunt	4.5	4.8% (4.3–5.3)	2.5% (1.6–3.5)
Railway	4.4	3.4% (2.9–3.8)	11.2% (9.3–13.1)
Other	6.6	6.7% (6.1–7.2)	6.6% (5.1–8.1)
Mode of transport (column % with 95% CI)			
Ambulance	39.8	37.4% (36.3–38.4)	57.1% (54.1–60.1)
Private car	27.5	29.5% (28.5–30.6)	13.5% (11.5–15.6)
Motor/Auto-rickshaw	15.9	17.1% (16.3–17.9)	8.1% (6.4–9.7)
Police	9.7	9.7% (8.9–10.3)	10.1% (8.3–11.9)
Walking	0.4	0.5% (0.3–0.6)	0.2% (0.1–0.5)
Public transport	0.2	0.2% (0.1–0.4)	0.2% (0.1–0.5)
Unspecified	5.7	5.1% (4.6–5.6)	10.5% (8.7–12.3)
Other	0.6	0.6% (0.4–0.8)	0.4% (0.1–0.7)
ISS—Median (IQR)	9 (4–14)	9 (4–13)	11 (9–18)

Variables	*n* (col %)	*n* (row %)	*n* (row %)
Age			
< 15 years	1447 (16.8)	1351 (93.4)	96 (6.6)
15–55 years	6220 (72.3)	5433 (87.3)	787 (12.7)
> 55 years	939 (10.9)	755 (80.4)	184 (19.6)
Injury Severity Score (ISS)			
< 9	2688 (94.0)	2861 (34.5)	173 (6.0)
9–15	3049 (86.8)	3514 (42.3)	465 (13.2)
16–25	1236 (80.8)	1530 (18.4)	294 (19.2)
> 25	301 (76.0)	396 (4.8)	95 (24.0)
Glasgow Coma Scale			
13–15	6429 (76.4)	6186 (96.2)	243 (3.8)
9–12	740 (8.8)	599 (80.9)	141 (19.1)
3–8	1241 (14.8)	575 (46.3)	666 (53.7)
Systolic BP (mmHg)			
≥ 90	8306 (96.5)	7377 (88.8)	929 (11.2)
< 90	300 (3.5)	162 (54.0)	138 (46.0)

Patients with a GCS ≤ 8 had a mortality rate of 53.7% (666 died / 1241 GCS ≤ 8). In patients with GCS ≥ 9, the mortality was 5.4% (384 died / 7169 GCS ≥ 9). (Fig. 2b) Shock on arrival (systolic blood pressure < 90 mm of Hg) was reported in 3.5% of all trauma patients, and the mortality in this subgroup was 46.0%. (Fig. 2c) Of 374 patients with railway injuries, 119 (31.0%) died; this was the most lethal mechanism of injury. This was followed by RTI, where 520/4032 patients (12.9%) died, and then falls in 2755 patients with 307 (11.1%) deaths. (Fig. 2d) Patients with a higher Injury Severity Score (ISS) > 25 had higher odds of mortality. Only 37% of patients survived up to 30 days among the most severely injured (ISS > 25) cases. (Fig. 2e).

Regarding the physiological parameters of SBP, HR, RR SpO2, and GCS, there was a statistically significant difference in the mean of each between survivors and non-survivors (*p* < 0.05). Compromised physiological parameters on arrival were seen more among non-survivors, as compared to that of survivors. The mean SBP of survivors (119.7 (16 mmHg)) was 5 mmHg higher than in non-survivors (114.8 (28 mmHg)). Similarly, non-survivors (101.2 (25)) had a mean first-recorded HR, which was 11 beats per minute higher than in survivors (91.1(16)). Further, similar differences in RR and oxygen saturation levels were recorded in those who survived and non-survivors (*p* < 0.001), as shown in Table 2.

**Table 2:** On-admission physiological and process-of-care parameters in trauma survivors and non-survivors in Indian urban hospitals

On-admission physiological parameters	Survivors *n*, Mean ± SD	Non-survivors *n*, Mean ± SD	*p* value	Missing data (%)
Systolic blood pressure (mm of Hg)	6913, 119.7 ± 16	948, 114.8 ± 28	< 0.001	8.7
Heart rate (beats per min)	7441, 91.1 ± 16	994, 101.2 ± 25	< 0.001	2.0
Respiratory rate (breaths per min)	6775, 19.3 ± 4.3	739, 20.4 ± 5.2	< 0.001	12.7
Oxygen saturation(%)^a^	100 (98–100)	99 (95–100)	< 0.001	17.9
Glasgow Coma Scale^a^	15 (15–15)	6 (3–12)	< 0.001	2.3

Process parameters (specified as HH:MM)	Median (IQR)^a^	Median (IQR)^a^	*p* value	Missing data (%)
Injury to hospital arrival	3:44 (1:11–13:02)	3:31 (1:00–12:33)	0.55	12.0
Hospital arrival to first vitals	0:18 (0:05–1:03)	0:24 (0:05–1:16)	< 0.001	26.3
Hospital arrival to admission	4:15 (1:47–9:43)	2:17 (0:47–5:23)	0.09	3.3
Injury to admission	9:00 (4:26–20:52)	6:09 (2:47–17:37)	0.39	4.3

^a^Values are median (interquartile range) unless otherwise indicated

Regarding the process of care measurements, the median time from: injury to arrival at hospital (*p* = 0.55), arrival to admission (*p* = 0.09), and injury to admission (*p* = 0.39) were not found to be statistically different between survivors and non-survivors. However, the 6-min difference in median time from arrival at the hospital to the first vital sign measurement, between survivors and non-survivors, was statistically significant (*p* < 0.001) (Table 2).

**Fig. 3:**
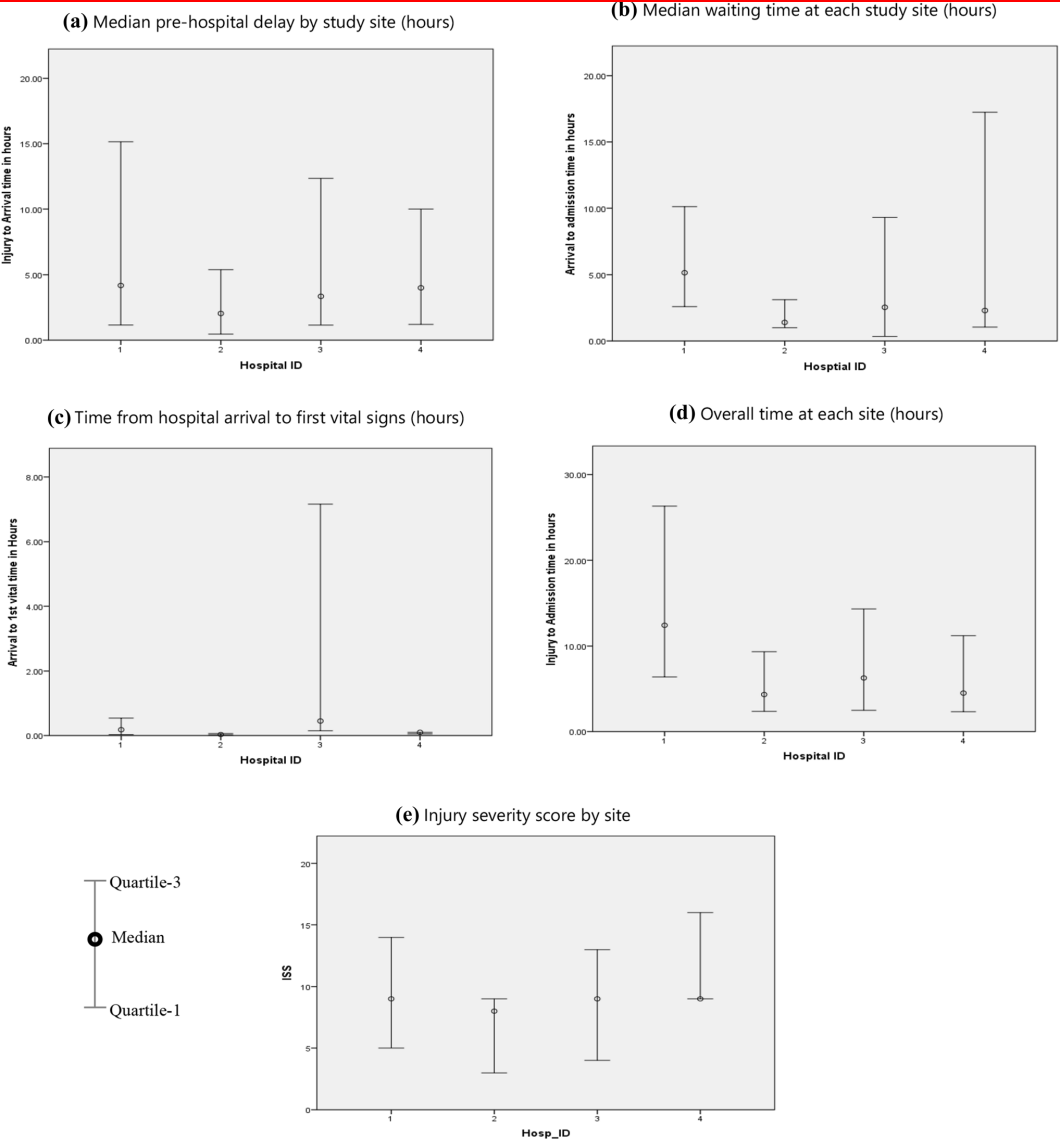
Process-of-care delays and Injury Severity Scores in trauma patients of four hospitals

24-Hour mortality was best predicted by the on-admission vital signs. However, as the length of in-hospital stay increased, the differences in on-admission vital signs between the survivors and the non-survivors decreased (Table 3) and, consequently, the ability of on-admission physiological vital signs and GCS to predict delayed and late mortality. The critical process of care indicator of the third delay (in receiving care), after arriving at many LMIC hospitals, is the time interval between the arrival of the trauma victim to the first vital measurement being taken (signaling the initiation of triage). The first vitals were measured much quicker in the survivors than non-survivors. We checked for the bias of missingness in this variable and found it evenly distributed in survivors and non-survivors. A subgroup analysis by delays in recording the first vital since arrival demonstrated that mortality increased after a 15-min delay (10.9% at ≤ 15 min to 15.0% ≥ 3 h). There were differences between the process-of-care parameters among the participating hospitals, and the case-mix also differed in each participating hospital (Fig. 3).

**Table 3:** Demographic, physiological, and process parameters in early, delayed, and late in-hospital trauma mortality (n = 8606)

Overall 30-day mortality 12.4% (Died *n* = 1067)	Early (24 h) mortality 1.9% (*n* = 164)		Delayed (1-– days) mortality 7.3% (*n* = 631)		Late (7–30 days) mortality 3.2%(*n* = 272)	
Variables	Survivors	Non-survivors	Survivors	Non-survivors	Survivors	Non-survivors
Mean Age (range)	29 (0–80)	34 (0–90)	28 (0–100)	36 (0–90)	34 (0–95)	41 (0–92)
Males	82.8%	89.6%	80.7%	81.5%	82.8%	82.0%
Females	17.2%	10.4%	19.3%	18.5%	17.2%	18.0%

On-admission physiological variable	Mean (SD)	Mean (SD)	Mean (SD)	Mean (SD)	Mean (SD)	Mean (SD)
Systolic blood pressure (mmHg)	121 (18)	105 (32)	119 (15)	115 (28)	120 (18)	118 (24)
Heart rate	94 (18)	105 (31)	90 (15)	102 (25)	92 (18)	98 (24)
Respiratory rate	20 (4)	21 (5)	19 (4)	21 (5)	19 (5)	20 (5)
Oxygen saturation %	98 (6)	88 (18)	99 (3)	94 (11)	99 (3)	96 (8)
Glasgow coma scale	14 (3)	6 (4)	14 (2)	7 (4)	13 (3)	10 (4)

Process parameters (HH:MM)	Median (IQR)	Median (IQR)	Median (IQR)	Median (IQR)	Median (IQR)	Median (IQR)
Injury to arrival (pre-hosp delay)	2:09 (0:50–7:00)	3:12 (1:00–11:30)	4:56 (1:30–16:10)	1:16 (0:40–3:60)	3:35 (1:00–11:30)	6:05 (1:50–21:20)
Arrival to first vital (treatment time)	0:10 (0:04–0:50)	0:20 (0:10–1:10)	0:17 (0:10–1:10)	0:23 (0:10–1:20)	0:18 (0:04–0:57)	0:30 (0:10–1:20)
Arrival to admission (waiting time)	1:20 (0:52–2:30)	4:16 (1:50–9:50)	4:44 (2:10–10:40)	1:10 (0:30–2:50)	2:19 (0:40–5:30)	3:21 (1:20–6:50)
Injury to admission (overall time)	4:07 (2:10–9:20)	8:07 (4:10–18:40)	11:22 (5:40–26:20)	3:00 (1:40–6:10)	5:58 (2:50–16:10)	10:09 (4:60–28:40)

## Discussion

This study examined the 30-day mortality rate in Indian urban trauma centers and estimated it to be 12.4% of all trauma admissions. This Indian rate was higher than the in-hospital mortality rates reported by HIC trauma centers for similarly injured patients [8, 9]. However, the current 12.4% mortality rate was an improvement over that previously reported by Indian trauma registries of 21.4% (2015) from similar urban trauma centers [6]. The 43% reduction in 30-day mortality is dramatic, as compared to the recent earlier studies. This reduction is more significant than most other trends (for example, reduction in maternal or infant mortality). There have been improvements in communications, development of infrastructure, and equipment. But there has been no significant trauma systems implementation or intervention (neither pre-hospital nor in-hospital) that can be credited for this observed improvement in mortality. Therefore, the authors would like to attribute this reduction to the differing case-mix between the previous studies and the current one. Our study sites were Indian urban referral trauma centers at University hospitals and are not representative of the broader situation across India [6, 12]

The majority of deaths are in the first week, but after the first 24 h, interventions need to be designed to address this subgroup of fatalities. The delayed deaths may be attributed to an inadequate pre-hospital resuscitation during the transfer from the injury site to the hospital, with no pre-hospital fluid or blood resuscitation during a hospital transfer. The low incidence of patients in hypovolemic shock (and barely recordable BP) on hospital arrival (3.5%) may suggest that many of the severely compromised patients who would have died within 24-h of in-hospital stay may have died in the pre-hospital phase. For physiological parameters (SBP, HR, RR, SpO2), statistically significant associations existed. However, this significant association may not be clinically relevant and such finding may be due to the high power (i.e., large sample size) of the study.

A low GCS (< 9) on arrival was seen to be associated with 53% mortality in our study. In many trauma studies, a low on-arrival GCS was considered as a strong predictor of trauma mortality [13–15]. Our data confirmed this finding and the overall mortality progressively decreased with an increasing on-arrival GCS. It reduced from 19% in the moderate GCS category to 3% in the mild GCS category. This finding of the GCS and mortality association in this large dataset validates the appropriate use of GCS for triage on-admission in Indian trauma patients. Increasing age is associated with a higher probability of 30-day mortality and this is consistent with findings worldwide [15].

The process of care delays tend to be relatively pronounced across LMICs, including India [6]. The ‘second delay’ or pre-hospital transit time was the median time between injury and arrival (the delay in reaching care, also called the second delay) was slightly longer for survivors than non-survivors. This may mean that although there is no formal system of pre-hospital triage, injury victims with severe conditions likely to die need to be sent directly to the trauma center by the first responder, who could be a bystander or the police [16].

The ‘third’ delay or the delay in initiating management is an important delay peculiar to the LMICs and has not been researched adequately. In our study, this was the median time from arrival at hospital to first measurement of vital signs, and this served as a proxy measure for the third-delay. The third delay was significantly shorter (*p* < 0.001) for survivors than non-survivors. Relative proportions of survival in each of the groups were compared based upon time to first vital sign recording. We found that delays greater than 15 min, as a proxy for the third delay, was seen in non-survivors. While it is tempting to attribute death to the longer third delay in non-survivors, this particular variable was missing in 26.3% of cases. However, this is an opportunity for improvement, as the patient had arrived at the trauma receiving center, but did not have their vitals checked immediately, resulting in a delayed triage. This is often attributed to the backlog of patients in the receiving area, a lack of pre-hospital notification, other on-going procedures in a human resource constrained environment and varying trauma care protocols [16].

This third delay could be improved by immediate triaging on arrival and pre-hospital notification and trauma team call-out protocols, which have been piloted in a separate arm of this AITSC research project. The variability in the process of care across different participating institutions is visible in the graphs. However, we did not compare between participating institutions as they had differing financial and manpower resources.

While the study’s strengths were that geographically diverse large-Indian cities were included in a registry with a relatively low proportion of missing values, this study had many limitations. The study design included only high-volume urban referral trauma tertiary-care centers, and therefore, the findings are externally valid only in the Indian urban setting. There is a referral bias as more severe cases are referred to the study hospitals, which leads to relatively higher mortality in tertiary care institutions [17]. Further, patients who were unable to reach a tertiary care hospital or died en route were not included in the AITSC registry or this study. Therefore, we are unable to comment on the pre-hospital care processes and outcomes in this paper. Perhaps some of the higher socioeconomic group patients and stable patients may have availed treatment in private institutions, and there is a bias toward the lower socioeconomic group trauma patients, who come to public university hospitals. This contributes to the pedestrian (road or rail) trauma victim pool in our registry, as compared to car-occupants.

Also, mortality after discharge (even within 30 days) could not be captured in this study, as there was no protocol of home-based follow-up in this study. Capturing time from documented notes is often inaccurate and injury time is often a matter of conjecture. Also, resuscitative treatment can often begin before actual documentation, especially in an unstable patient. In India, admission time varies with the administrative formalities for issuing an admission case file.

The implications of this study are that a standard measure is used to measure mortality across Indian trauma centers, allowing comparison across trauma centers. On-admission physiological vital signs are adequate triage tools for prioritizing patients who are likely to die within 24 h. The best practices and reduced time delays in the best performing participating sites can be replicated across other institutions to improve survival in trauma victims.

Besides pre-hospital notification, the AITSC project also studied improved trauma reception and resuscitation, trauma quality improvement programs, and post-trauma discharge rehabilitation [9]. The findings of these arms once published will augment the knowledge base of this research paper. Mortality remains a gross measure of outcome, and further research into trauma morbidity would be important.

## Conclusion

One in ten admitted trauma patients (12.4%) died in urban trauma centers in India. More than half of the trauma deaths were delayed, beyond 24 h but within a week. Further research is needed to determine the causes of these deaths. Physiological vital signs remain a valid predictor of early 24-h trauma mortality but were less predictive of late and delayed mortality. Early initiation of trauma assessment and monitoring immediately on arrival was important to predict 30-day survival.
